# Deciphering Protein–Protein Interactions. Part II. Computational Methods to Predict Protein and Domain Interaction Partners

**DOI:** 10.1371/journal.pcbi.0030043

**Published:** 2007-04-27

**Authors:** Benjamin A Shoemaker, Anna R Panchenko

**Affiliations:** Whitehead Institute, United States of America

Recent advances in high-throughput experimental methods for the identification of protein interactions have resulted in a large amount of diverse data that are somewhat incomplete and contradictory. As valuable as they are, such experimental approaches studying protein interactomes have certain limitations that can be complemented by the computational methods for predicting protein interactions. In this review we describe different approaches to predict protein interaction partners as well as highlight recent achievements in the prediction of specific domains mediating protein–protein interactions. We discuss the applicability of computational methods to different types of prediction problems and point out limitations common to all of them.

## Introduction

In our companion review published in the last issue [1], we outlined the experimental techniques for the identification and characterization of protein interactions. We showed that high-throughput experimental methods produce a large amount of data which needs to be analyzed and verified. Despite this, interactomes of many organisms are far from complete. The low interaction coverage along with the experimental biases toward certain protein types and cellular localizations reported by most experimental techniques call for the development of computational methods to predict whether two proteins interact. These methods can be very useful for choosing potential targets for experimental screening or for validating experimental data (see [1]) and can provide information about interaction details (in the case of domain prediction methods) which might not be apparent from the experimental techniques. Many methods use combinations of experimental and computational techniques to different extent (for example, gene co-expression and synthetic lethality methods were covered among experimental approaches in our companion paper [1]) and do not predict physical interactions directly but rather infer the functional associations between potentially interacting proteins.

In this review, we report on several methods to predict protein or domain interaction partners. Some computational methods are based on the co-localization of potentially interacting genes in the same gene clusters or protein chains (gene cluster, gene neighborhood, and Rosetta stone methods), on co-evolution patterns in interacting proteins (sequence co-evolution methods), and on the co-expression of genes. Some methods find patterns of co-occurences in interacting proteins, protein domains, and phenotypes (phylogenetic profiles and synthetic lethality methods), while others use the presence of sequence/structural motifs characteristic only for interacting proteins (classification methods, association methods). To analyze interaction specificity at the domain level, in this second paper of the review we describe methods that are aimed at identifying specific domains mediating interactions in an interacting protein pair.

## Methods for Predicting Protein Interaction Partners

**Figure 1:**
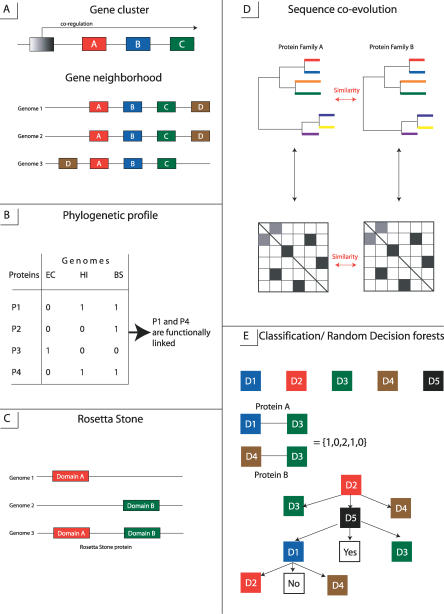
Different Methods of Protein Interaction Prediction (A) Gene cluster and gene neighborhood methods, different boxes showing different genes. (B) Phylogenetic profile method, showing the presence/absence of four proteins in three genomes. (C) Rosetta Stone method. (D) Sequence co-evolution method looking for the similarity between two phylogenetic trees/distance matrices (E) Classification methods shown with the example of RFD method, where five different features/domains are used and each interacting protein pair is encoded as a string of 0, 1, and 2. The decision trees are constructed based on the training set of interacting protein pairs and decisions are made if proteins under the question interact or not (“yes” for interacting, “no” for non-interacting).

Table 1 lists different protein interaction methods, and Figure 1 illustrates their main ideas. We start the review with the genomic inference methods [2] (gene neighbor, gene cluster, Rosetta stone, and phylogenetic profile) that use genomic/protein context to infer functional associations. Gene neighbor and gene cluster methods are referred to as GN.

**Table 1:**
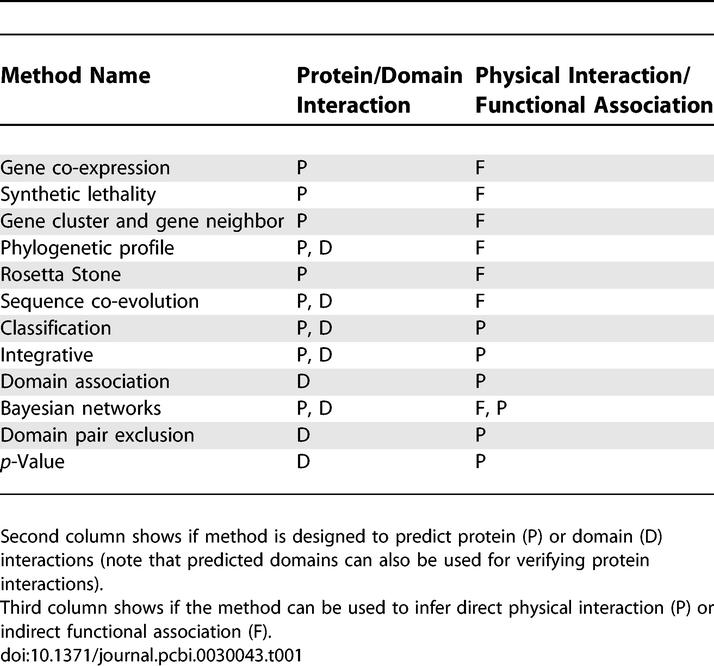
Different Prediction Methods

### 

#### Gene neighbor and gene cluster methods.

Genes with closely related functions encoding potentially interacting proteins are often transcribed as a single unit, an operon, in bacteria and are co-regulated in eukaryotes. Different methods have been developed trying to predict operons based on intergenic distances [2–6] (Figure 1A). Despite the effect of neutral evolution which tends to shuffle gene order between distantly related organisms, gene clusters or operons encoding for co-regulated genes are usually conserved; and operons found by gene neighbor methods can provide additional evidence about functional linkage between their constituent genes [2,7–10]. Analysis of gene order conservation within three bacterial and archaeal genomes found that 63%–75% of co-regulated genes interact physically [7,11]. Similar results were obtained from two eukaryotes, yeast and worm [12]. Moreover, it was found that GN methods have higher coverage (about 37%) compared with other genomic inference methods [11]. An interesting example of GN involves the prediction of archael exosome by comparing gene order in archaeal and eukaryotic genomes [13]. The predicted archaeal exosomal superoperon was confirmed later by the experiment [14] and was shown to encode among other proteins two protein subunits of RNase P. This suggested a possible interaction between RNase P and the exosome in eukaryotes, a connection that was not reported earlier.

#### Phylogenetic profile methods.

The phylogenetic profile (PP) method is based on the hypothesis that functionally linked and potentially interacting nonhomologous proteins co-evolve and have orthologs in the same subset of fully sequenced organisms [9,15–19]. Indeed, components of complexes and pathways should be present simultaneously in order to perform their functions. A phylogenetic profile is constructed for each protein, as a vector of *N* elements, where *N* is the number of genomes (Figure 1B). The presence/absence of a given protein in a given genome is indicated as “1” or “0” at each position of a profile. Proteins or their profiles can then be clustered using a bit-distance measure, and those proteins from the same cluster are considered functionally related. Higher-order relationships between several proteins also can be identified using extensions of PP [20,21]. Phylogenetic profiles can also be identified for protein domains instead of entire proteins [22]. A profile is constructed for each domain and the presence/absence of the domain in different genomes is recorded which in turn can give information about domain interactions.

Some drawbacks of PP include its high computational cost, its dependence on high information profiles, and homology detection between distant organisms. For example, ubiquitous unlinked proteins present in all genomes (profiles with all “1”s) will be counted by PP as correlated. The same is true for proteins that are specific to a given genome (profiles with all, but one, “0”s). Shared phylogenetic relationships between two proteins can also produce false correlations between profiles. This issue has recently been addressed by incorporating the phylogenetic trees in the analysis of correlated gains and losses of pairs of proteins [23].

#### Rosetta Stone method.

The Rosetta Stone approach infers protein interactions from protein sequences in different genomes [24–27]. It is based on the observation that some interacting proteins/domains have homologs in other genomes that are fused into one protein chain, a so-called Rosetta Stone protein (Figure 1C). Gene fusion apparently occurs to optimize co-expression of genes encoding for interacting proteins. In *Escherichia coli,* the Rosetta Stone method found 6,809 potentially interacting pairs of nonhomologous proteins; both proteins from each pair had significant sequence similarity to a single protein from some other genome. Analysis of pairs found by this approach revealed that for more than half of the pairs both members were functionally related [24]. Comparison with the experimental data on protein interactions from the DIP database showed that about 6.4% of all experimental interactions can be linked by Rosetta Stone proteins.

#### Sequence-based co-evolution methods.

As was mentioned earlier, interacting proteins very often co-evolve so that changes in one protein leading to the loss of function or interaction should be compensated by the correlated changes in another protein. The orthologs of coevolving proteins also tend to interact, thereby making it possible to infer unknown interactions in other genomes [28]. It has been argued that co-evolution can be reflected in terms of the similarity between phylogenetic trees of two non-homologous interacting protein families (Figure 1D). The similarity between phylogenetic trees can be quantified by calculating the correlation coefficient between distance matrices used to construct the trees with large values indicating co-evolution between two protein families [29,30] or domain families [31]. Correspondence between the elements of two matrices or branches of two trees is required to calculate the correlation coefficient, but such information is not always available. To address this issue, several algorithms have been developed to identify specific interaction partners between two interacting families that are especially useful when families contain paralogs with different binding specificities [32–34]. Given a pair of protein families, their distance matrices are aligned to minimize the difference between their elements, and interactions are predicted as those corresponding to aligned columns of two matrices. It was noticed earlier that most methods cannot perform an alignment search successfully if the size of families is large (more than 30 proteins in a family) [32]. One way to reduce the search space is to use the information encoded in phylogenetic trees [34].

The similarity between two phylogenetic trees is influenced by the speciation process, and therefore there is a certain “background” similarity between trees of any proteins, no matter if they interact or not. Different statistical techniques have been developed to account for “phylogenetic subtraction” [35]. Simplified versions of this approach were introduced recently to account for the background similarity in protein interaction prediction [36–38]. According to one of them [36], the “background” tree is constructed from the 16S rRNA sequences and is considered to be a canonical tree of life. The final distance matrices are obtained by subtracting the rescaled rRNA-based distances from the evolutionary distances obtained from the original phylogenetic trees. It has been shown that this method finds 50% of real interacting proteins at a 6.4% false positive rate compared with the 16.5% false positive rate obtained using methods which do not take into account evolutionary distances and the “background” canonical tree [29,30].

One example of how co-evolution studies could be used in confirming and predicting putative interaction partners is the case of DNA colicins and their immunity proteins [39]. Colicins consist of an N-terminal domain participating in translocation across the membrane of the target cell, the central domain which specifically binds to the extracellular surface receptor, and the C-terminal domain responsible for the toxic activity of colicin. Each DNase colicin has a specific immunity protein, which binds to the toxic domain and inhibits its cytotoxic activity. Co-evolution studies showed that there is a significant correlation between the two families of DNA colicins and their immunity proteins (with the correlation coefficient of 0.67), with weaker correlation between Im2, Im8, and Im9 immunity proteins and their corresponding binding partners. Experimental studies indicated that there is indeed a cross-reactivity between colicin E9 and Im8 and Im2 proteins [40].

#### Classification methods.

Different classification methods have been successfully applied to the prediction of protein and domain interactions [41–54]. These methods use various data sources to train a classifier to distinguish between positive examples of truly interacting protein/domain pairs from the negative examples of non-interacting pairs. Kernel methods are particularly useful in this respect as they provide a vectorial representation of data in the feature space through the set of pairwise comparisons [54]. Each protein or protein pair can be encoded as a feature vector where features may represent a particular information source on protein interactions, domain compositions, or evidence coming from various experimental methods. As a result of a comparison of different classifiers, it has been shown that Random Forest Decision (RFD) consistently ranks as a top classifier, with Support Vector Machines being in second place [55].

Figure 1E shows an example of the use of RFD to predict protein interactions. RFD builds decision trees based on the domain composition of interacting and non-interacting proteins, explores all possible combinations of interacting domains, and predicts at the end if a given pair of proteins interacts [43]. Each protein pair is represented as a vector of length N, where N is the number of different domain types (features), and each feature can have values 2, 1, or 0 depending if this domain is found in both proteins, in one of them, or not found in the protein pair. Given an experimental training set of interacting protein pairs, the method constructs a decision tree (or many trees) which defines the best splitting feature at each node from a randomly selected feature subspace. The best feature is selected based on the measure of “goodness of fit,” which estimates how well this feature can discriminate between two classes of interacting and non-interacting pairs. The method stops growing the tree as soon as all pairs at a given node are well-separated into two classes. Traversing along the tree provides a classification for an unknown protein pair.

Multiple sources of direct and indirect data on protein–protein interactions can be combined in a supervised learning framework or integrative scoring scheme to predict protein–protein and domain–domain interactions [47,53,56–60]. It has been shown that the prediction accuracy is improved when several sources of data are used, and, in addition, integrative approaches can provide means to justify the confidence of inferred interactions.

## Predicting Domain Interactions from Protein Interactions

By far the most coverage of experimental data describing protein interaction networks comes from high-throughput experiments giving us the identity of interacting protein pairs (see our previous review [1]). Unfortunately, these experiments reveal no structural details about the interaction interfaces and the formation of protein complexes. To deal with these limitations, several approaches have been developed to predict which domains in a protein pair interact given a set of experimental protein interactions; some of them focus on interactions involving specific mediating domains/peptides (SH2, SH3, PDZ domains) [61,62].

The following section gives an overview of domain prediction methods which are listed in Table 1. Note that some of the approaches already mentioned for protein interaction prediction, namely the sequence co-evolution, phylogenetic profiles, and classification methods, are also applicable to domain interaction prediction. Most methods begin by annotating protein sequences with domains that can be defined by Pfam, SCOP, CDD, or other domain databases [63–65]. The methods are typically trained on high-throughput protein interaction data. Predicted domain interactions are evaluated using structural data or by higher quality interaction sets such as MIPS [66]. Moreover, accounting for domains in proteins and domain interaction networks can in turn help in predicting protein interactions [67–70].

### 

#### Association methods.

**Figure 2:**
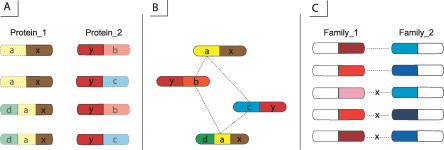
Strategies to Predict Domain Interactions from Protein Interactions (A) Shows that due to the abundance of domains *x* and *y* in protein interaction pairs shown on the same line, the domains *x* and *y* are predicted to interact. (B) Illustrates the same dataset revealing that the actual domain interactions (dotted lines) do not include domains *x* and *y*. It shows that accounting for other domains in a protein pair in addition to *x* and *y* can result in alternative predictions. (C) Considers the case of several paralogous protein pairs (from Family_1 and Family_2) containing the same two domains. In this case each paralog from one domain family (represented by a shade of red for Family_1) interacts with only one specific paralog (represented by a shade of blue for Family_2) of the other domain family. While there are examples of specific interacting domains (shown by dotted line), there are even more cases where they do not interact (shown with an “X”), meaning that the larger abundance of non-interacting examples can mask the few, specific interacting cases.

This group of methods looks for the characteristic sequence or structural motifs which distinguish interacting proteins from non-interacting [71–74]. For this purpose association methods can use different classifiers (see previous section), and some of them are tuned specifically to identify domains responsible for protein interactions. For example, as shown in Figure 2A, correlated sequence signatures, or domains, that are found together more often than expected by chance can be used as markers to predict a new type of protein interaction [71]. In this case, protein interaction data is used to compute log-odds scores and to find correlated domains. The log-odds score is computed as: log_2_(*P_ij_/P_i_P_j_*), where *P_ij_* is the observed frequency of domains *i* and *j* occurring in one protein pair; *P_i_* and *P_j_* are the background frequencies of domains *i* and *j* in the data. In this approach predicted domain interactions have been defined as those having positive log-odds scores and having several instances of occurrence of a given domain pair in the database. Using this method, it was found that certain domains can be found quite often in protein interacting pairs and can be used for protein interaction prediction.

#### Bayesian network models and maximum likelihood methods.

The association method which uses correlated sequence signatures [71] considers each pair of interacting domains separately, ignoring other domains in a given pair of interacting proteins (Figure 2A and 2B). Moreover, many association methods do not explicitly take into account the missing and incorrect interaction data which can be treated by using the Bayesian network methods [67,75,76]. To estimate the parameters of Bayesian models, the Maximum Likelihood Estimation method (MLE) [76] can be used. MLE maximizes the probability of interaction of all putative domain pairs and incorporates the experimental errors of protein interaction data into the scoring scheme (Figure 2B). The likelihood function is a function of parameters *θ(λ_ij_, f_p_, f_n_)*, where *λ_ij_* is the probability that domains *i* and *j* interact, *f_p_* is the false positive rate, and *f_n_* is the false negative rate derived from experimental data. It is difficult to maximize the likelihood function directly because of the large number of parameters (large number of different types of interacting domains). To solve this problem, the Expectation Maximization algorithm is used to find maximum likelihood estimates of unknown parameters *θ* by finding the expectation of the complete data consisting of observed data and unobserved data in two iterative steps. The observed data includes protein–protein interactions and the domain composition of the proteins, and the unobserved data includes all putative domain–domain interactions.

#### Domain pair exclusion analysis.

The domain pair exclusion analysis method extends the previously described MLE method and can detect specific domain interactions (see Figure 2C) which are hard to detect using MLE [77]. MLE and other methods emphasize nonspecific promiscuous domain interactions which are detected as those having large *θ* values. On the contrary, specific, rare interactions between certain members of two domain families can be neglected. The domain pair exclusion analysis method accounts for this by estimating an *E_ij_* score which measures the evidence that domains *i* and *j* interact and is defined as the logarithm of a ratio of two probabilities. The numerator corresponds to the probability that two proteins interact given that domains *i* and *j* interact. The denominator corresponds to the probability that proteins interact given that domains *i* and *j* do not interact. To compute E-scores for a given domain pair, the probability in the numerator is calculated with the Expectation Maximization procedure (similar to the one described in the previous section). For the probability in the denominator, the procedure is repeated where the probability for a given pair of domains to interact is set to zero. This allows the competing domains to maximize *θ_ij_*.

A high E-score value shows the high propensity of two domains to interact, while a low value indicates that competing domains from the same protein pair are more likely to be responsible for this interaction. Therefore, specific domain interactions can be found by screening for low *θ* values and high E-scores. Although this model does not account for false positives and negatives in the experimental data, it was shown that the E-scores perform better than its constituent quantities, finding 2.9 times more true positives than random assignment; for comparison, *θ* values yield 1.4 times more true positives than random assignments [77] .

#### *p*-Value method.

The *p*-value method tests a null hypothesis that the presence of a particular domain pair in a protein pair has no effect on whether two proteins interact [78]. To test this hypothesis, a statistic is calculated for each domain pair which takes into account experimental error (fraction of false positives) and incompleteness of the dataset (fraction of false negatives). The reference distribution is simulated by shuffling domains in proteins so that the network of protein interactions remains fixed. Obtained *p*-values show the reliability of domain interactions given that two proteins interact, and the domain pair with the lowest *p*-value is most likely to interact. The *p*-value method performs reasonably well when there are nine or more domains on a protein pair. However, interestingly enough, for the majority of test cases, random domain prediction outperforms all methods tested, pointing to the low accuracy of all prediction methods of domain interactions.

The methods of domain interaction prediction described in this section all have varying degrees of success, but have limitations common to most of them. First, domains are assumed to interact independently, although their interactions can depend on other domains in a protein pair. Second, incomplete domain assignments, due to insufficient coverage of domain databases and limited searching ability of domain profiles, can lead to false positive and negative interaction predictions. Finally, protein interaction data is not complete, whereas domain prediction methods are based on this data.

In this paper, we reviewed various computational methods to predict protein and domain interaction partners [79]. All of these methods use experimental data sources, some of them to a larger extent (gene co-expression, synthetic lethality) than others. As a result, they all suffer from the limitations of experimental approaches and incompleteness of observed data. Despite the fact that there is a certain circularity in testing the computational methods on experimental data, their prediction accuracy proved to be increasing, which makes them useful for the validation and analysis of diverse protein interactomes. The majority of presented prediction methods do not rely on protein structures and potentially can be applied on the genome-wide scale; while structural analysis can provide further details of protein–protein and domain–domain interfaces and give clues on their modeling. 

## Abbreviations

GN: gene neighbor and gene cluster
MLE: Maximum Likelihood Estimation
PP: phylogenetic profile
RFD: Random Forest Decision
